# Fine-Needle Aspiration Cytology of Ewing's Sarcoma of Thoracic Spine with Extension into the Intradural Space

**DOI:** 10.1155/2014/351386

**Published:** 2014-02-03

**Authors:** Sandhya Bordia, Sweta Meena, Bijendar Kumar Meena, Vijay Rajak

**Affiliations:** ^1^Department of Pathology, RNT Medical College, Udaipur, Rajasthan 313001, India; ^2^Department of Radiodiagnosis, RNT Medical College, Udaipur, Rajasthan 313001, India

## Abstract

Ewing's sarcoma/peripheral primitive neuroectodermal tumor is a small, round, and blue cell malignancy that occurs most often in bone and soft tissues of children and young adults. The intraspinal manifestation of the disease is rare, and when present, this is often misdiagnosed with other varieties of primary spinal tumors. Fine-needle aspiration cytology (FNAC) plays important role in the early diagnosis of these cases. We report such a case of Ewing's sarcoma of thoracic spine with extension into the intradural space, which was initially suspected to be a case of metastatic lesion in an 18-year-old boy.

## 1. Introduction

Ewing's sarcoma family of tumors are a group of small round-cell neoplasms, which include Ewing's sarcoma (EWS), primitive neuroectodermal tumor (PNET), Askins tumor, PNET of the bone, and extraosseous Ewing's sarcoma (ESS) [1]. Ewing's sarcoma/peripheral primitive neuroectodermal tumor is presumed to be in neuroectodermal children and young adults. The intraspinal extraosseous Ewing's sarcoma (CNS-ESS) is extremely rare and often misdiagnosed.

## 2. Case Report

An 18-year-old boy was admitted to Neurosurgery Ward of RNT Medical College, Udaipur Rajasthan, with chief complaints of progressively increasing dullaching pain in the lower back and both limbs and tenderness at D7, D8 vertebral region. There was a history of loss of appetite and malaise. The patient was thin built and on general examination there was a mild pallor. Systemic examination did not reveal any abnormalities except weakness (power 3/5) in both lower limbs and decrease in sensation of touch.

**Figure 1:**
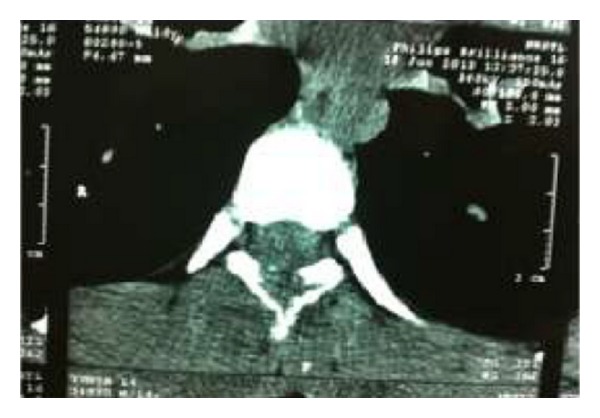


Investigation revealed haemoglobin 9 gm.%, total leukocyte count 8200 cells/mm cu with 70% polymorphs and 26% lymphocytes. Erythrocyte sedimentation rate was 60 mm at the end of 1 hour. The urine examination and bone marrow examination did not reveal any abnormality. Tuberculin test was negative. Chest radiograph was normal. CT scan (Figure 1) of the spine showed an expansile lytic lesion of posterior element of D7 vertebrae involving the spinous process, associated with heterogeneously enhancing soft tissue component, which was extending from D7 to D9 vertebrae.

**Figure 2:**
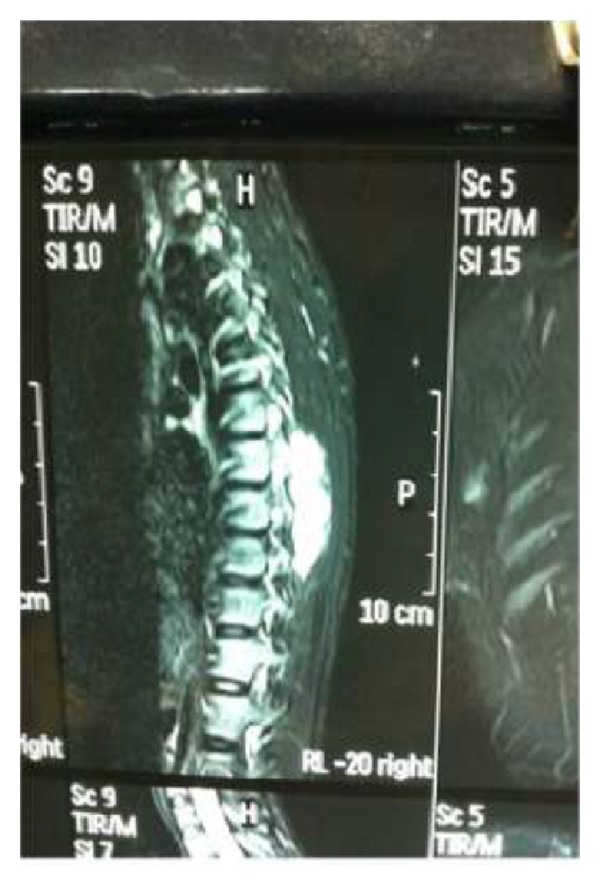


**Figure 3:**
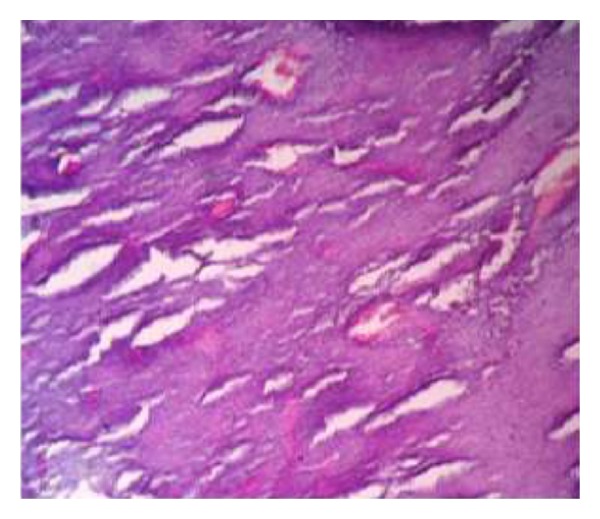


**Figure 4:**
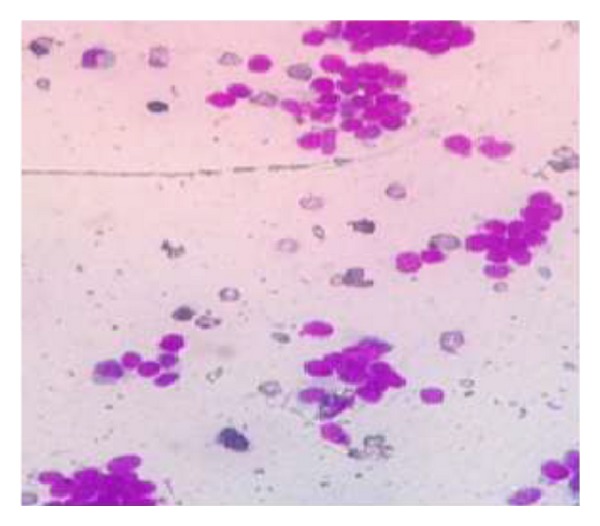


**Figure 5:**
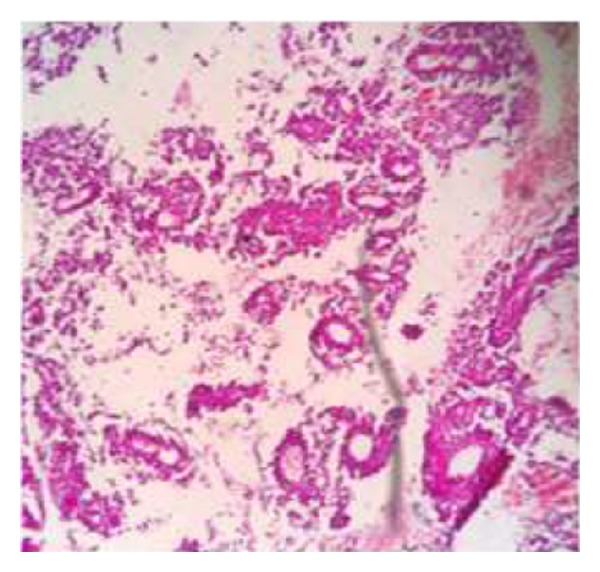


**Figure 6:**
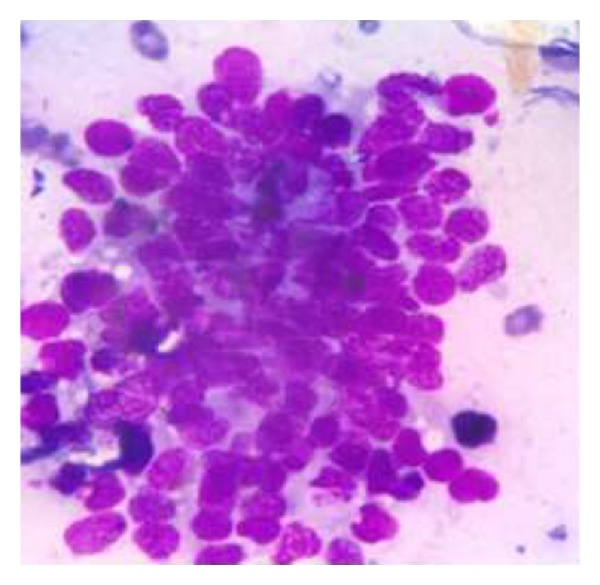


The soft tissue component was seen involving the thecal sac, lamina, and spinous process of D7 and causing compression and displacement of spinal cord anteriorly. MRI of the spine (Figure 2) reveals an expansile lesion of posterior element of D7 involving spinous process and neural arch, associated with fairly large, enhancing soft tissue component {67∗32∗18} in posterior paraspinal region extending from D6-D7 to D8-D9 levels, extending into epidural compartment of canal at D6-7 and D7-8, and causing compression and displacement of spinal cord anteriorly with severe secondary central canal stenosis AP diameter approximately 2 mm. FNAC was advised for definite diagnosis. USG guided FNAC was done using long 22-gauge disposable needle and 10 mL syringe. Slides were prepared and stained with May-Grunwald-Giemsa (MGG). Two slides were reserved for special stain and studies. FNAC revealed cellular smear composed of densely dispersed, small, monomorphic round cells with fine nuclear chromatin and round nuclei and scanty clear cytoplasm. Many cells showed irregularly vacuolated cytoplasm. Occasional rosette formation was also seen (Figures 4 and 6). FNAC diagnosis of malignant small, round-cell tumor, most likely Ewing's sarcoma, was offered. Histopathological section revealed small blue round cell with monomorphic appearance. Pseudorosettes were also noted and vascularization was prominent (Figure 5). PAS stain revealed positive intracellular deposit of glycogen (Figure 3). The patient was referred to radiotherapy ward for further management; immunohistochemistry was performed there in which the tumor cells exhibited NSE and Vimentin along with diffuse membranous staining for CD-99.

## 3. Discussion

James Ewing described the first case of ES in 1921 [1]. Ewing's sarcoma is usually seen in the age group of 5–30 years with a peak incidence at 10–15 years. The male-to-female ratio is 3 : 2. Although it can involve any bone, it is more common in the bones of the lower extremity. In the vertebral column, sacral involvement dominates followed by the lumbar, thoracic, cervical, and coccygeal regions in order of decreasing frequency [2]. The dorsal vertebrae are involved in 1% of cases [3]. Ewing's sarcoma is highly malignant round-cell tumor and has aggressive clinical behaviour and distant metastasis. CT scan and MRI are essential for establishing the extraskeletal site of tumor [4]. Cytology and histopathology are essential for definite diagnosis. ES is richly vascular and shows an abundance of thin walled vessels within fibrovascular septae.

ES and pPNET both are the two ends of the spectrum which differ in morphology as well as neuronal differentiation. This family of tumors share common cytogenetic and molecular changes which involve the translocation of Ewing's sarcoma gene on chromosome 22 (22q12) onto chromosome 11 (11q24). The tumor also shares expression of glycoprotein surface antigen mic2/CD99 [5].

Differential diagnosis includes round-cell tumors like metastatic neuroblastoma, rhabdomyosarcoma, and non-Hodgkin lymphoma (NHL). Skeletal Ewing's sarcoma is known to have a 5-year survival of 75%. The EES and pPNET on the other hand have 38% survival [6].

## 4. Conclusion

FNAC is a very economic and quick procedure in the diagnosis of Ewing's sarcoma family of tumors. Accurate diagnosis can be made in deep seated tumor as in our case by adequate material under radiological guidance. FNAC is also useful in long term followup [7].

## References

[1] Yadav TP, Singh RP, Gupta VK, Chaturvedi NK, Prasad CV (1998). Paravertebral extra osseous Ewing’s sarcoma. *Indian Pediatrics*.

[2] Resnik D, Kyriakas M, Greenway GD, Resnik D (1995). Tumour and tumour like lesions of bone, imaging and thology of specific lesions. *Diagnosis of Bone and Joint Disorders*.

[3] Greenfield GB, Greenfield GB Primary malignant tumours of bone. *Radiology of Bone Diseases*.

[4] Kaspers G-J, Kamphorst W, van de Graaff M, van Alphen AM, Veerman AJP (1991). Primary spinal epidural extraosseous Ewing’s sarcoma. *Cancer*.

[5] de Alava E, Kawai A, Healey JH EWS-FLI1 fusion transcript structure is an independent determinant of prognosis in Ewing's sarcoma. *Journal of Clinical Oncology*.

[6] Rosai J, Rosai A (2009). Bone and joints. *Surgical Pathology*.

[7] Kilpatrick SE, Geisinger KR (1998). Soft tissue sarcomas: the usefulness and limitations of fine-needle aspiration biopsy. *The American Journal of Clinical Pathology*.

